# Impaired Autophagy in Krabbe Disease: The Role of BCL2 and Beclin-1 Phosphorylation

**DOI:** 10.3390/ijms24065984

**Published:** 2023-03-22

**Authors:** Nadia Papini, Roberta Todisco, Paola Giussani, Michele Dei Cas, Rita Paroni, Chiara Giallanza, Cristina Tringali

**Affiliations:** 1 Department of Medical Biotechnology and Translational Medicine, Università degli Studi di Milano, 20054 Segrate, MI, Italy; 2 Department of Health Sciences, Università degli Studi di Milano, 20142 Milan, Italy

**Keywords:** Krabbe disease, autophagy, BCL2, beclin-1

## Abstract

Autophagic impairment was identified in many lysosomal storage diseases and adult neurodegenerative diseases. It seems that this defect could be directly related to the appearance of a neurodegenerative phenotype and could contribute to worsen metabolite accumulation and lysosomal distress. Thus, autophagy is becoming a promising target for supportive therapies. Autophagy alterations were recently identified also in Krabbe disease. Krabbe disease is characterized by extensive demyelination and dysmyelination and it is due to the genetic loss of function of the lysosomal enzyme galactocerebrosidase (GALC). This enzyme leads to the accumulation of galactosylceramide, psychosine, and secondary substrates such as lactosylceramide. In this paper, we induced autophagy through starvation and examined the cellular response occurring in fibroblasts isolated from patients. We demonstrated that the inhibitory AKT-mediated phosphorylation of beclin-1 and the BCL2-beclin-1 complex concur to reduce autophagosomes formation in response to starvation. These events were not dependent on the accumulation of psychosine, which was previously identified as a possible player in autophagic impairment in Krabbe disease. We believe that these data could better elucidate the capability of response to autophagic stimuli in Krabbe disease, in order to identify possible molecules able to stimulate the process.

## 1. Introduction

In all its forms (macroautophagy, selective autophagy, chaperone-mediated autophagy, and microautophagy), autophagy is deeply involved in nervous system development and functioning. Many processes strictly depend on autophagy, namely the removal of proteins that could form aggregates if present in excess and the preservation of axons. Furthermore, the engagement of Schwann cells in myelination, remyelination, myelin debris clearance after damage, neurogenesis, and the maintenance of adult neural stem cells, as extensively reviewed by Menzies et al. [1], are also linked to autophagy. Accordingly, autophagy dysfunctions were recognized in many neurodegenerative diseases, including Alzheimer’s and Parkinson’s diseases, amyotrophic lateral sclerosis, and polyglutamine disorders [1]. Autophagy impairment was also identified in many lysosomal storage diseases which most frequently are associated with neurodegeneration in children [2]. In these diseases, autophagy dysfunctions were usually linked to lysosomal damage. The damage consists of alterations of membrane lipids which, in turn, modify autophagosome-lysosome fusion, in the inadequate lysosomal acidification and in the altered trafficking of hydrolytic enzymes. Eventually, lysosomal damage alters the functionality of the transcription factor EB (TFEB), which controls lysosomal biogenesis [1,2,3].

By now, in Krabbe disease (KD), also known as globoid cell leukodystrophy (OMIM #245200), several proofs of autophagy impairment were reported and mostly explained by the accumulation of psychosine (PSY) (galactosylsphingosine) that is characteristic of the disease and caused by the deficiency of the lysosomal enzyme galactocerebrosidase (GALC). Firstly, after adding PSY, Ribbens et al. identified increased cleavage levels of microtubule-associated protein light chain (LC3) 1B in the immortalized neuroglial cell line, 145M-Twi, established from twitcher mouse brain [4]. Then, PSY was demonstrated to enhance autophagic flux in the human oligodendrocyte cell line MO3.13 [5]. p62/sequestome 1 (SQSTM1) levels increased, if compared with wild-type (WT) murine strains, in brain sections, sciatic nerve, and fibroblasts isolated from twitcher mouse, suggesting autophagy impairment [6]. It was proved that while brief administration of PSY induced autophagy, a long exposure to high doses of this molecule caused the impairment of autophagy and of the ubiquitin-proteasome system (UPS), leading to the accumulation of cytoplasmic aggregates composed of ubiquitin and p62/SQSTM1 and visible in white matter and spinal cord of twitcher mice [7]. In agreement with the evidence of alterations in autophagy, aggregates of alpha-synuclein, a typical autophagic target, were recognized in twitcher mice and KD patients [8,9,10].

In this view, several studies tested the autophagic response induced by lithium and demonstrated that lithium increased the survival of MO3.13 oligodendrocytes treated with PSY [5] and transiently ameliorated muscle strength in twitcher mice. On the contrary, in vivo, autophagy was not differently induced, at least at the doses and timing used for lithium administration by the authors [11].

Autophagy is a promising topic to be better explored in KD to find novel therapeutic tools to improve patients’ survival and well-being. Nevertheless, the molecular mechanisms that cause autophagy dysfunction in KD disease should be still fully elucidated before they can be used as therapeutic targets. Although PSY accumulation plays a key role in this process, some evidence suggests that this molecule by itself cannot account for all defects detected in autophagy. In particular, it emerged that fibroblasts isolated from twitcher mice showed signs of autophagy dysfunction even if endogenous PSY was present at levels comparable to the ones present in WT fibroblasts, without PSY added exogenously [6].

Recently, we demonstrated that human KD fibroblasts are characterized by increased levels of lactosylceramide (LacCer), a substrate of GALC. In turn, LacCer is responsible for the altered activation of several signalling molecules, among which the most relevant are AKT and B-cell lymphoma 2 (BCL2), which are both upregulated [12]. Since the AKT pathway and BCL2 were shown to interact with the autophagic machinery [13,14,15], we investigated the effects of starvation to trigger autophagy in KD human fibroblasts which, as we previously reported, did not accumulate PSY [12]. Our results demonstrated that, independently from PSY, KD fibroblasts showed several defects in the autophagic response and that they can be associated with AKT upregulation.

## 2. Results

### 2.1. Autophagosomes Formation Is Reduced in KD Fibroblasts under Starvation

**Figure 1:**
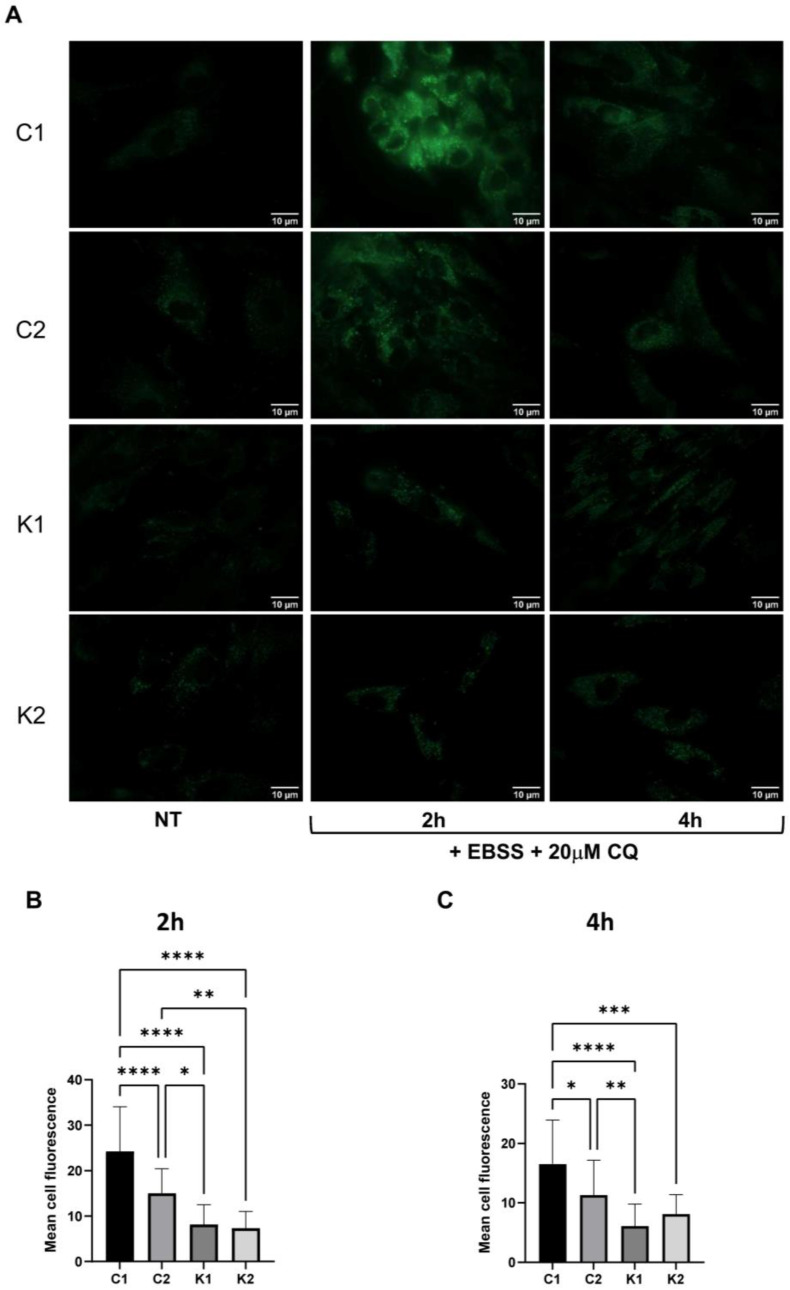
(**A**) Representative images of fibroblasts stained with MCD after 2 h and 4 h in EBSS plus 20 μM CQ captured through an Olympus BX-50 fluorescence microscope at 100× objective (oil immersion). (**B**) Mean cell fluorescence determined after 2 h in EBSS plus 20 μM CQ and (**C**) after 4 h. Fluorescence was measured in all cells present in six different fields. Statistical significance was calculated by one-way ANOVA and Tukey’s multiple comparisons tests *: *p* < 0.05; **: *p* < 0.01; ***: *p* < 0.001; ****: *p* < 0.0001. C1: Control 1 fibroblasts; C2: Control 2 fibroblasts; K1: Krabbe 1 fibroblasts; K2: Krabbe 2 fibroblasts.

Experiments were performed using L40 and RB1818 fibroblasts isolated from children not affected by KD (referred to as Control 1 and Control 2, respectively, or as control fibroblasts) and VA1679 and FO86/78 fibroblasts isolated from KD patients (referred to as Krabbe 1 and Krabbe 2, respectively, or as KD fibroblasts). To assess the response of KD fibroblasts to autophagic stimuli, we induced starvation by cultivating cells in Earle’s balanced salt solution (EBSS) for 2 h and 4 h. To better appreciate autophagosomes formation, we simultaneously added chloroquine (CQ) to block degradation. CQ alkalinizes the lysosomal lumen [16] and impairs autophagosome-lysosome fusion [17]. The employment of this inhibitor allows a better analysis of the issue. Autophagosomes were firstly stained using monodansylcadaverine (MDC). As shown in Figure 1, in basal conditions of growing, no appreciable differences or significant formation of autophagosomes were revealed in all fibroblasts. Above all, after 2 h upon starvation, autophagosomes were significantly more abundant in control than in KD fibroblasts. After 4 h, this trend was confirmed even if to a lower extent. To confirm these results, we performed the same experiment by maintaining cells in starvation with CQ and by staining cells using the Cyto-ID Green detection reagent. This reagent was reported to be a cationic amphiphilic dye specific for autophagosomes and with minimal staining of lysosomes. As shown in Figure S1, we confirmed that autophagosome formation induced by starvation was enhanced in control fibroblasts after 2 h of starvation.

### 2.2. The Autophagic Response Is Impaired in KD Fibroblasts

To investigate autophagic response in KD fibroblasts, we induced autophagy by culturing cells in EBSS for 2 h and 4 h and we examined the levels of several proteins involved in this process.

**Figure 2:**
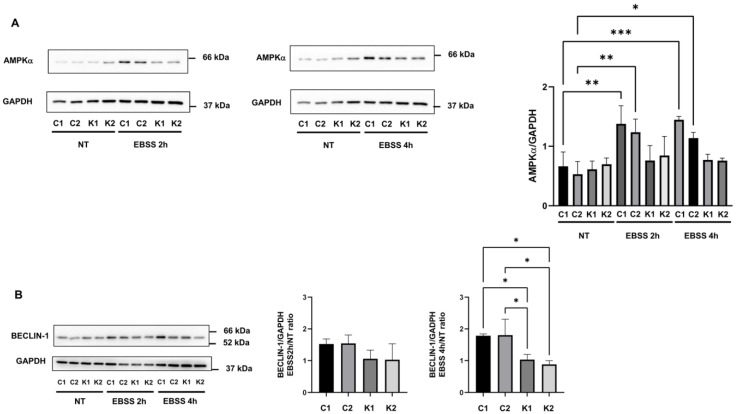
Representative Western blotting images and densitometric analysis of (**A**) AMPKα and (**B**) beclin-1 in growth conditions (NT) and after incubation with EBSS for 2 h and 4 h. Beclin-1 densitometric values are expressed as the ratio between EBSS 2 h or 4 h and growth conditions. Data are the mean ± SD of independent experiments (*n* = 3). GAPDH was employed as a loading control. Statistical significance was calculated by one-way ANOVA and Tukey’s multiple comparisons tests *: *p* < 0.05; **: *p* < 0.01; ***: *p* < 0.001. C1: Control 1 fibroblasts; C2: Control 2 fibroblasts; K1: Krabbe 1 fibroblasts; K2: Krabbe 2 fibroblasts. NT: non-treated.

A condition of serum starvation or nutrient deprivation such as EBSS starvation causes the activation of the AMP-activated protein kinase (AMPK), which initiates autophagy [18,19]. The analysis of AMPK phosphorylation at Thr172, required for the activation of this enzyme [19], did not reveal significant differences across the conditions (data not shown). Conversely, we observed a significant increase in AMPK content in control fibroblasts at 2 h and 4 h after autophagy induction in comparison with non-treated conditions (Figure 2A). In KD fibroblasts, AMPK content did not raise during autophagy (Figure 2A).

Then, we examined the expression of beclin-1, which is a key autophagy protein acting during the nucleation stage [20]. It was reported that in autophagy, beclin-1 expression is regulated at the transcriptional level [20]. As shown in Figure 2B, we found a higher increase in beclin-1 4 h after the induction in autophagy in control fibroblasts, compared to KD fibroblasts.

One of the most prominent indirect methods to study autophagic activity in cells is the measurement of LC3-II levels [21]. During autophagy, LC3 proteins are cleaved at the C-terminal amino acids to expose a glycine residue forming LC3-I and, subsequently, are conjugated to phosphatidylethanolamine (PE). This leads to their association with the internal and external surface of autophagosomes [22]. Levels of LC3-II in absence and in presence of lysosomal activity inhibitors allow us to determine whether the autophagic flux is activated or blocked. In particular, in presence of a lysosomal inhibitor such as CQ, an accumulation of LC3-II is considered a marker of autophagy flux activation [21,23,24].

**Figure 3:**
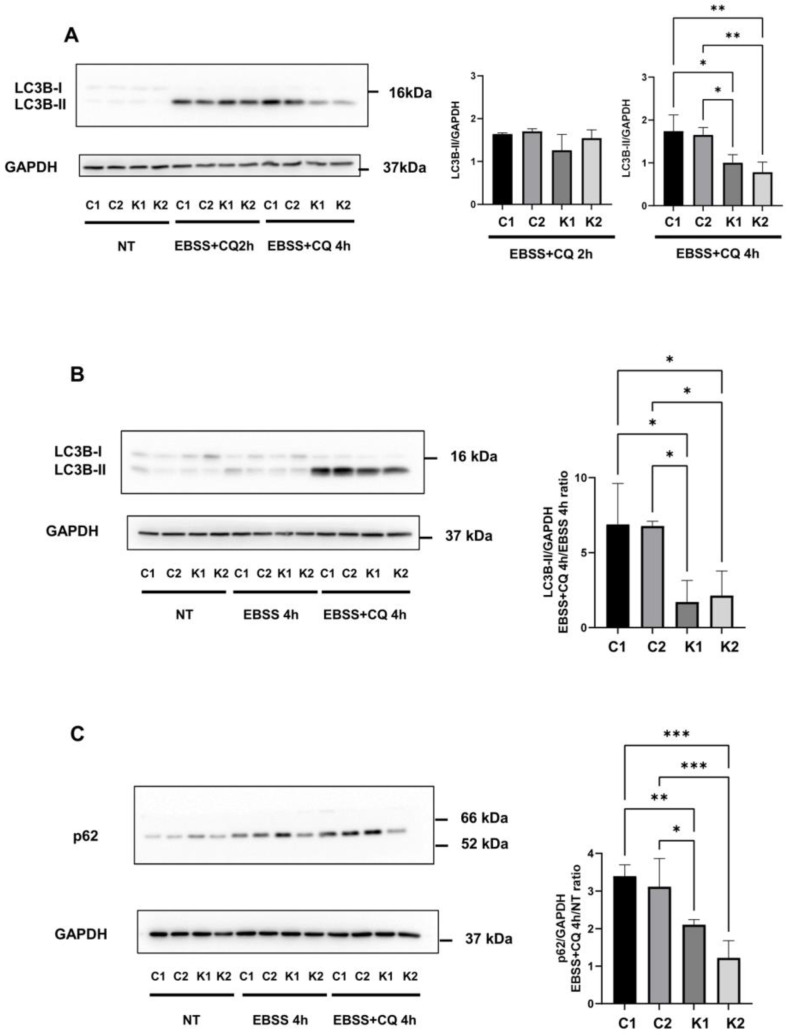
(**A**) Representative Western blotting images of LC3B-I and LC3B-II proteins in growth conditions (NT) and after incubation in EBSS with CQ for 2 h and 4 h and densitometric analysis of LC3B-II accumulation after incubation in EBSS with CQ for 2 h and 4 h. Data are reported as mean ± SD of independent experiments (n = 3). (**B**) Representative Western blotting image of LC3B-I and LC3B-II proteins in growth conditions (NT) and after incubation with EBSS and CQ for 4 h. Densitometric analysis of LC3B-II accumulation is calculated as a ratio between EBSS-CQ 4 h and EBSS 4 h conditions. Data are mean ± SD of independent experiments (*n* = 3). (**C**) Representative Western blotting image of p62/SQSTM1 protein in growth conditions (NT) and after incubation with EBSS and CQ for 4 h. Densitometric analysis of p62/ SQSTM1 is calculated as a ratio between EBSS + CQ 4 h and NT. Data are mean ± SD of independent experiments (*n* = 4). GAPDH was employed as a loading control. Statistical significance was calculated by one-way ANOVA and Tukey’s multiple comparisons tests *: *p* < 0.05; **: *p* < 0.01; ***: *p* < 0.001. C1: Control 1 fibroblasts; C2: Control 2 fibroblasts; K1: Krabbe 1 fibroblasts; K2: Krabbe 2 fibroblasts. NT: non-treated.

To study the autophagic flux we analysed by Western blotting the levels of LC3B-II in EBSS in absence and presence of 20 μM CQ for 2 h and 4 h. LC3B is the most studied isoform of the LC3 family [25]. As shown in Figure 3A, by cultivating cells in EBSS with CQ for 4 h, we observed a major accumulation of LC3B-II in normal fibroblasts than in KD fibroblasts. LC3B-II appeared to not increase further after 4 h of culture in EBSS with CQ in comparison to 2 h of culture in EBSS with CQ and in KD fibroblasts, particularly, it seemed to decrease. This effect could be possibly explained by hypothesizing that LC3B-II could also be partially lost through other routes such as the exosome release related to autophagy induction, as previously demonstrated [26,27]. However, given these problematics arose considering only LC3B-II formation and to better clarify LC3B-II behaviour, we calculated the ratio between normalized LC3B-II content in cells treated in EBSS with CQ and LC3B-II content in cells in EBSS without CQ, in order to monitor the autophagic flux. We found that in KD fibroblasts, LC3B-II content was lower than in normal fibroblasts (about 3–4-fold) (Figure 3B).

The decrease in the autophagic flux in KD fibroblasts was also corroborated by analysing p62/SQSTM1 levels during autophagy in presence of CQ compared to non-treated conditions. p62 /SQSTM1 is an ubiquitin-binding scaffold protein involved in ubiquitin-proteasome and autophagy degradation processes and it is used as an autophagic marker [21,28]. As shown in Figure 3C, the ratio between p62 levels in EBSS and in presence of CQ for 4 h and growth conditions was significantly higher in normal fibroblasts than in KD fibroblasts. Thus, in KD fibroblasts, there was a minor accumulation of p62/SQSTM1 in presence of the inhibitor of autophagosome degradation. Surprisingly, we did not observe a decrease in p62 (p62/SQSTM1) levels in cells cultivated in EBSS without CQ for 4 h (Figure 3C).

### 2.3. Lysosomal Alterations during Starvation-Induced Autophagy in KD Fibroblasts

**Figure 4:**
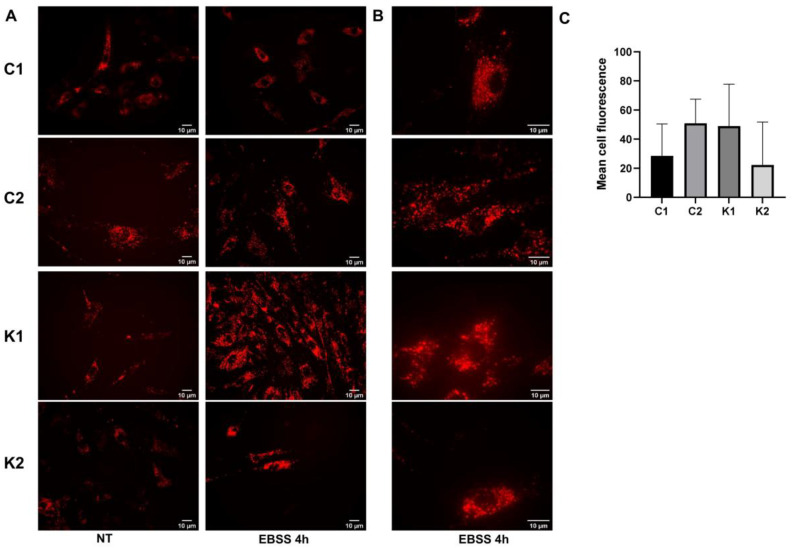

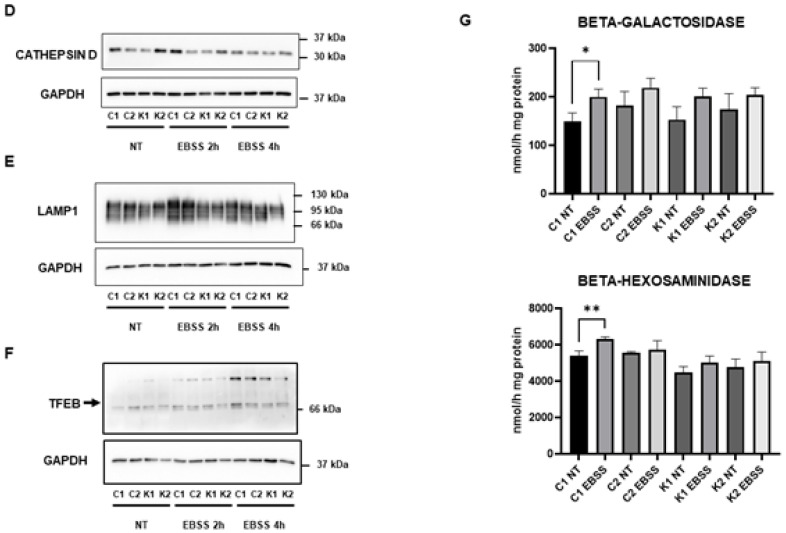
Representative images of fibroblasts stained with Lysotracker Red DND-99 after 4 h in EBSS, acquired using an Olympus BX-50 fluorescence microscope at (**A**) 40× objective and (**B**) 100× objective (oil immersion). (**C**) Mean cell fluorescence determined after 4 h in EBSS. Fluorescence was measured in all cells present in six different fields captured with 100× objective. Representative Western blot images of (**D**) cathepsin D (**E**) LAMP1, and (**F**) TFEB in growth conditions (NT) and after incubation in EBSS for 2 h and 4 h. (**G**) beta-galactosidase and beta-hexosaminidase activities measured in growth condition (NT) and after 4 h in EBSS. Data are the mean ± SD of independent experiments (*n* = 3). Statistical significance was calculated by unpaired t-Student comparing activity recorded after culture in EBSS (EBSS) with that recorded in growth condition (NT) for each cell type. *: *p* < 0.05; **: *p* < 0.01. C1: Control 1 fibroblasts; C2: Control 2 fibroblasts; K1: Krabbe 1 fibroblasts; K2: Krabbe 2 fibroblasts. NT: non-treated.

To investigate if lysosomes showed some dysfunctions during starvation-induced autophagy, we stained them using the LysoTracker probe. After 4 h of culture in EBSS, we did not detect any differences in the mean cell fluorescence among the four types of fibroblasts (Figure 4A–C). Examining cells at a higher magnification, we could better appreciate that LysoTracker staining evidenced dot structures in control fibroblasts after EBSS treatment; on the other hand, in KD fibroblasts, fluorescence appeared to be more diffused and possibly related to the phenotype of dilated lysosomes (Figure 4B). We did not detect a specific increase in cathepsin D, LAMP1 and TFEB expression in all fibroblasts during starvation (Figure 4D–F). To better investigate lysosomal functionality, we determined beta-galactosidase and beta-hexosaminidase enzymatic activities before and after 4 h in EBSS. We did not identify any changes except for the increase in both enzymatic activities in Control 1 cells after starvation. Despite that, this evidence was not confirmed in Control 2 cells (Figure 4G). Thus, based on our data, we were not able to identify specific alterations in lysosomal behaviour during autophagy induction in KD fibroblasts.

### 2.4. Cell Sphingolipids and Autophagy

**Figure 5:**
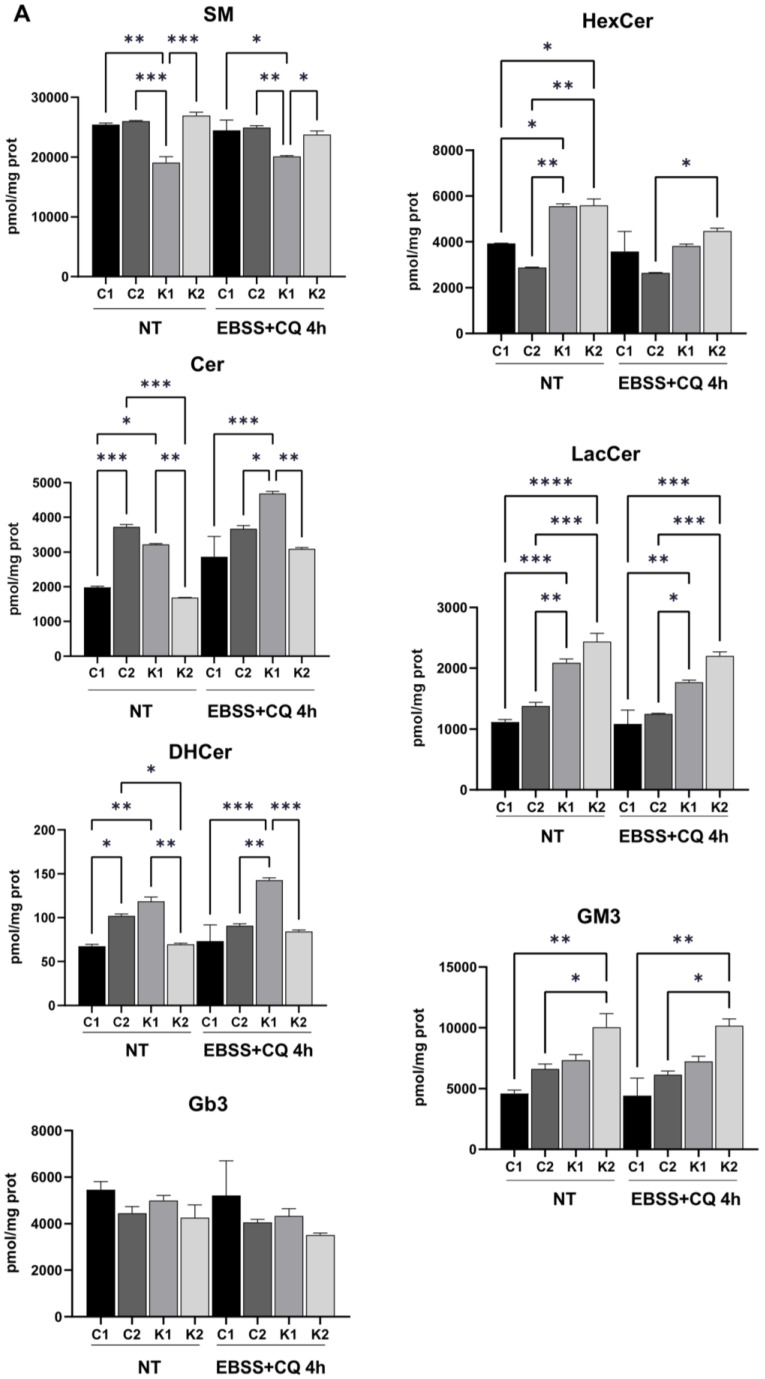

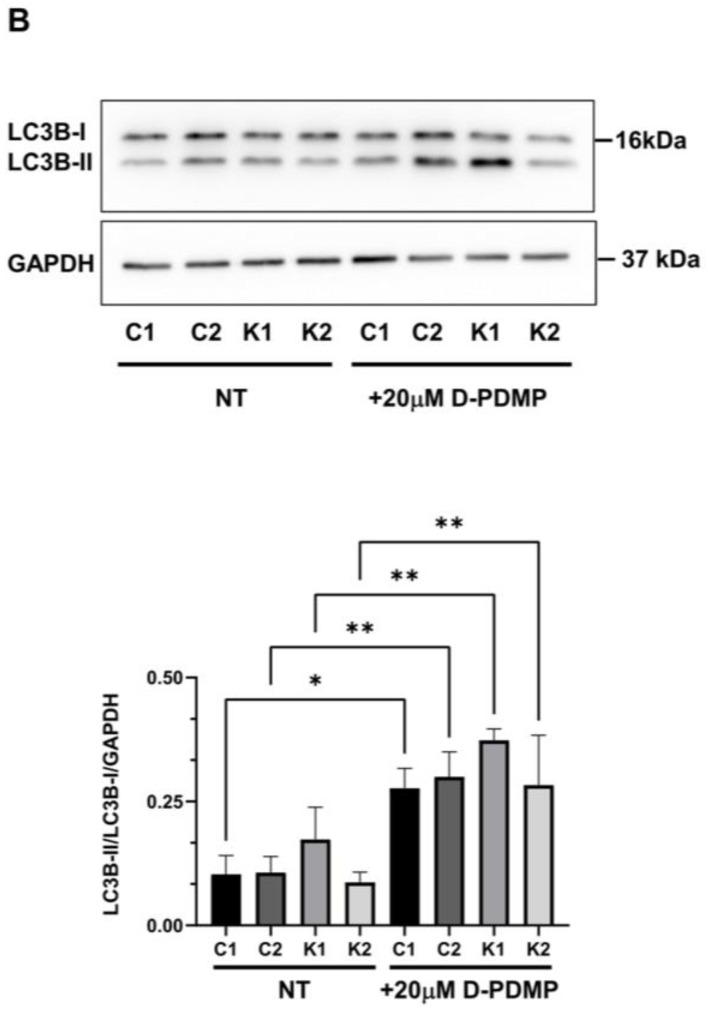
(**A**) Cell sphingolipid analysis performed in growth condition (NT) and after 4 h in EBSS plus 20 µM CQ. Statistical significance was determined through one-way ANOVA and Bonferroni’s multiple comparisons (*n* = 2). *: *p* < 0.05; **: *p* < 0.01; ***: *p* < 0.001; ****: *p* < 0.0001. SM: sphingomyelin; Cer: ceramide; DHCer: dihydroceramide; Gb3: globoside 3; HexCer: hexosylceramide; LacCer: lactosylceramide. (**B**) Representative Western blot LC3B-I and LC3B-II proteins in growth conditions (NT) and after treatment with 20 µM D-PDMP for 24 h. Densitometric analysis of LC3B-II/LC3B-I ratio. Data are mean ± SD of independent experiments (*n* = 3). Statistical significance was calculated by one-way ANOVA and Tukey’s multiple comparisons tests *: *p* < 0.05; **: *p* < 0.01. C1: Control 1 fibroblasts; C2: Control 2 fibroblasts; K1: Krabbe 1 fibroblasts; K2: Krabbe 2 fibroblasts. NT: non-treated.

We previously identified the increase in LacCer in KD fibroblasts [12], in absence of PSY accumulation. Thus, we explored cell sphingolipid profiles during starvation to understand if changes could occur. The analysis was performed through LC-MS/MS. We confirmed that PSY was not present in KD fibroblasts [12] and that it was not formed also during starvation with CQ. Regarding the other sphingolipids, we compared their content in control fibroblasts with the one in KD fibroblasts in both conditions (without treatment and under starvation plus CQ). Sphingomyelin (SM) content was lower in Krabbe 1 cells than in both control fibroblasts and Krabbe 2 fibroblasts before and after 4 h in starvation and CQ. Ceramide (Cer) and dihydroceramide (DHCer) appeared to increase in KD fibroblasts after 4 h in starvation and CQ, but only in Krabbe 1 fibroblasts they were significantly different if compared to control fibroblasts. Hexosylceramide (HexCer) significantly increased in KD fibroblasts before starvation coherently to the possible impairment of galactosylceramide (GalCer) catabolism. Nevertheless, HexCer decreased in both KD fibroblasts after starvation with CQ, possibly because of a reduction in its synthesis. LacCer levels increased in both KD fibroblasts in comparison to controls before and after 4 h of starvation. Globoside 3 (Gb3) and ganglioside GM3 levels did not change significantly (Figure 5A).

Thus, we can conclude that altered levels of LacCer were the main hallmark in cell sphingolipid profile to distinguish KD fibroblasts from controls before and after autophagy induction by starvation.

We further treated control and KD fibroblasts with d-threo-1-phenyl-2-decanoylamino-3-morpholino-1-propanol (D-PDMP) for 24 h. D-PDMP is an inhibitor of glucosylceramide synthetase and thus, it blocks glycosphingolipid synthesis. Additionally, it was demonstrated that D-PDMP is able to induce autophagy by increasing Cer levels [29] and by inhibiting mTORC1 [30]. This treatment induced LC3BII formation in control and KD fibroblasts at similar levels (Figure 5B), suggesting that the inhibition of glycosphingolipid synthesis and mTORC1 could be efficacious in stimulating autophagy in KD fibroblasts.

### 2.5. Involvement of Beclin-1 and BCL2 in Autophagy Impairment

**Figure 6:**
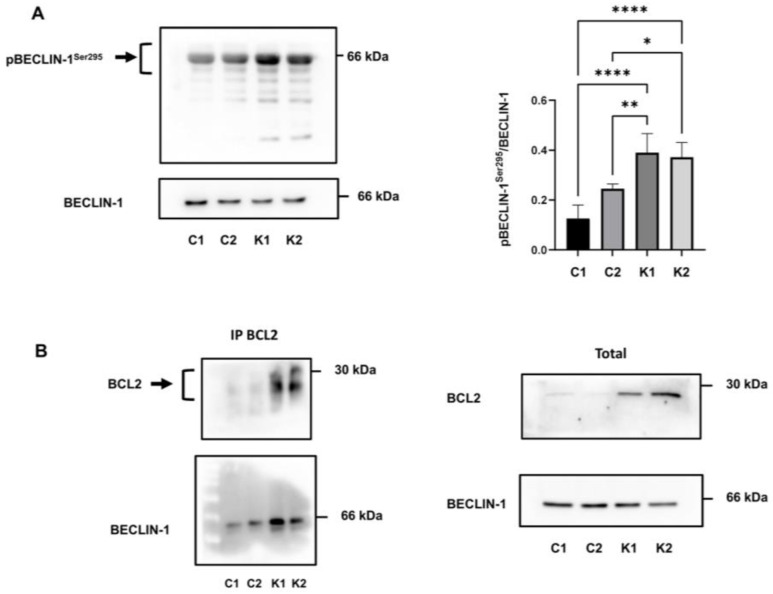
(**A**) Representative immunoblot image and densitometric analysis of p(Ser295)beclin-1 and beclin-1. Phosphorylated protein is plotted as the ratio of phosphorylated versus total protein. Data are reported as mean ± SD of independent experiments (*n* = 4). Statistical significance was calculated using one-way ANOVA and Tukey’s multiple comparisons tests. *: *p* < 0.05; **: *p* < 0.01; ****: *p* < 0.0001. (**B**) Immunoprecipitation of beclin-1 using an anti-BCL2 antibody. Representative images of immunoprecipitated proteins (left) and total extract (right) analysed by Western blotting using the indicated antibody (*n* = 3). C1: Control 1 fibroblasts; C2: Control 2 fibroblasts; K1: Krabbe 1 fibroblasts; K2: Krabbe 2 fibroblasts.

It is known that beclin-1 plays an important role in autophagy and it is regulated by different factors and mechanisms [31]. One of these mechanisms acting at the post-translational level is beclin-1 phosphorylation. Beclin-1 can be phosphorylated at different sites leading to activation or inhibition of this protein; in particular, AKT-mediated phosphorylation on Ser295 inhibits beclin-1 activation [31,32,33]. Firstly, we examined the beclin-1 phosphorylation status on Ser295 in growth conditions to understand the alterations of autophagy in KD fibroblasts. As shown in Figure 6A, KD fibroblasts had a significantly higher level of phosphorylation, compared to control fibroblasts.

The formation of the BCL2-beclin-1 complex is another pathway that regulates autophagy [34,35]. Since we noticed an increased expression of BCL2 in KD fibroblasts in a previous study [12], we investigated if beclin-1 was more bound with BCL2 in KD than in control fibroblasts and then less available for autophagy. To this purpose, we performed immunoprecipitation experiments in growth conditions using a BCL2 antibody. Immunoprecipitated protein complexes, analysed by Western blotting and by using antibodies against BCL2 and beclin-1, revealed a higher level of beclin-1 bound to BCL2 in KD fibroblasts than in control fibroblasts (Figure 6B). Specifically, we found that the 48%, 58%, 86%, and 84.4% of beclin-1 was bound to BCL2 in C1, C2, K1, and K2 fibroblasts, respectively (calculated as bound beclin-1/bound beclin-1 + unbound beclin-1).

**Figure 7:**
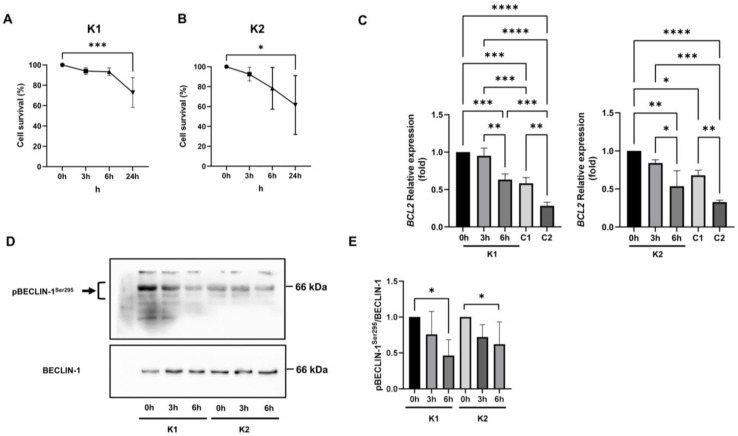
(**A**) Krabbe 1 (K1) and (**B**) Krabbe 2 (K2) cell survival upon 20 µM LY294002 treatment. Viable cells were stained with calcein AM. Significance was calculated using the ordinary one-way ANOVA test followed by Dunnett’s multiple comparisons test. *N* = 4 (for Krabbe 1 cells); *n* = 5 (for Krabbe 2 cells); ***: *p* < 0.001; *: *p* < 0.05. (**C**) BCL2 mRNA expression assessed by Real-Time PCR in Krabbe 1 (K1) and Krabbe 2 (K2) cells treated for 0 h, 3 h, and 6 h with 20 µM LY294002 and in Control 1 (C1) and Control 2 (C2) cells. Significance was calculated using the ordinary one-way ANOVA test followed by Tukey’s multiple comparisons test. *N* = 3; *: *p* < 0.05. **: *p* < 0.01; ***: *p* < 0.001; ****: *p* < 0.0001. (**D**) Representative Western blot images and (**E**) densitometric analysis of *p*(Ser295) beclin-1 normalized on beclin-1 expression in Krabbe 1 (K1) and Krabbe 2 (K2) fibroblasts treated for 0 h, 3 h, and 6 h with 20 µM LY294002. Significance was calculated using the ordinary one-way ANOVA test followed by Dunnett’s multiple comparisons test. *N* = 4; *: *p* < 0.05.

To assess if AKT upregulation could be responsible for the increase in Ser295 phosphorylation on beclin-1 observed in KD fibroblasts, we treated VA1679 and FO86/78 cells with the phosphatidylinositol 3-kinase (PI3K) inhibitor LY294002. To check viability, we performed the experiment for 3 h, 6 h, and 24 h and stained viable cells using calcein AM. We recognized a significant decline in cell survival only after 24 h of treatment in both cell lines (Figure 7A,B); thus, we decided to monitor the effects of LY294002 for 3 h and 6 h. We confirmed AKT inhibition by reviewing AKT Ser473-phosphorylation (data not shown). We assessed the decrease in BCL2 expression, significant only after 6 h of treatment, and confirmed that in both Krabbe 1 and Krabbe 2 cells, AKT upregulation played a pivotal role in controlling BCL2 expression (Figure 7C). After 6 h of treatment, BCL2 expression decreased to levels comparable to that of Control 1 cells (for Krabbe 1 cells) and Control 1 and Control 2 cells (for Krabbe 2 cells) (Figure 7C). BCL2 expression levels were recognized as a key factor in autophagic induction [35]. Then, we demonstrated that AKT inhibition promoted a significant decrease in p(Ser295) beclin-1 (Figure 7D,E), proving that AKT upregulation was considerably involved in beclin-1 inhibition in KD fibroblasts.

## 3. Discussion

A large consensus was established to consider substrate accumulation as a pivotal step of lysosomal storage disorders’ pathogenesis but, possibly, it is not the unique alteration responsible for the multiple facets of these diseases. The appearance of secondary anomalous metabolites, perturbations of signalling pathways, trafficking, metabolism, organelles functionality, and cellular processes, such as autophagy, have a great pathogenic drive [2,36]. Impaired autophagy was identified as a common dysfunction in many lysosomal storage diseases characterized by neurodegeneration. Autophagy dysfunction is deeply related to the appearance of neurodegenerative symptoms, as demonstrated by studies performed in Atg5 or Atg7 deficient mice. In addition, autophagy alterations could be involved in substrate accumulation because many molecules such as cholesterol and lipids are removed by this pathway, when functioning. Furthermore, aged or damaged mitochondria are renewed through autophagy and, for this reason, autophagic defect could increase oxidative stress [37]. In this perspective, some small molecules able to rescue autophagic pathways such as rapamycin, carbamazepine, and lithium are under investigation [37]. Autophagy inducers appear to be particularly promising if combined with other therapeutic options such as enzyme replacement therapy (ERT) [37]. Along this line, lithium administration was tested to improve the survival of PSY-treated MO3.13 oligodendrocytes [5].

We previously identified an increase in the nuclear fraction of the nuclear factor erythroid 2-related factor 2 (NRF2), which could be indicative of autophagic dysfunction, in human KD fibroblasts [12]. In fact, the increase in p62/Keap1 complexes could induce a prolonged activation of NRF2 [38,39]. Moreover, we demonstrated that in KD fibroblasts some signalling perturbations coexist; in particular, AKT activation and BCL2 expression are upregulated due to LacCer increase [12]. It is well-known that both AKT and BCL2 can act as autophagy modulators [40]. However, in normal growing conditions, we were not able to identify marked alterations in autophagosomes formation or in autophagic markers in human KD fibroblasts. Thus, we decided to better investigate the autophagic machinery by inducing autophagy through starvation.

This paper demonstrated an impaired autophagic response to starvation in KD fibroblasts. We first observed that the overall formation of autophagosomes appeared to be reduced. Since we excluded that this finding could be caused by the presence of PSY in KD fibroblasts [12], we investigated the molecular machinery involved in autophagy.

AMPK is a cellular energy sensor, and it is activated by starvation. It induces autophagy by phosphorylating ULK1, which is a promoter of autophagosomes formation [41] and which is involved in autophagosome maturation and lysosomal degradation [42]. During starvation, AMPK increased only in control cells, not in KD fibroblasts. Previously, it was reported that PSY reduced AMPK activity in oligodendrocytes and the investigation of AMPK activators in KD was proposed [43]. Overall, AMPK is activated in conditions that cause a reduction in ATP levels, such as starvation. Lysosome constitutes a critical compartment in AMPK activation signalling [44]; thus, it can be hypothesized that alterations in lysosomal functionality could have consequences on AMPK signalling. Our data demonstrated a defect in the response to energetic stress. This alteration consists of the increase in AMPK expression rather than in the activation/phosphorylation of AMPK. Thus, it is difficult to argue if the phosphorylating regulation of AMPK is impaired in KD fibroblasts. We supposed that AKT upregulation could influence the response to energetic stress induced by starvation in KD fibroblasts. The increase in AMPK expression that appeared to occur in starved control fibroblasts and that, on the contrary, was impaired in KD fibroblasts could be a consequence of this upregulation. Nevertheless, we can conclude that the AMPK response to starvation in KD fibroblasts was altered.

Beclin-1 is a key player during the early autophagic steps (initiation) and it is crucial for autophagosomes formation [45]. Beclin-1 can associate with different proteins such as Atg14L to activate the kinase Vps34 and Ambra1. It can also induce the aggregation of beclin-1-Vps34-Vps15 complexes that trigger autophagosomes formation [20]. During starvation, the increase in beclin-1 was significantly lower in KD fibroblasts than in control fibroblasts. LC3B protein processing is a common method used to monitor the autophagic flux. LC3B is cleaved by Atg4 to form LC3B-I, which is in turn associated with phosphatidylethanolamine to constitute LC3B-II. LC3B-II is crucial for autophagosomes formation; in fact, it is a key component of their membrane, and it is degraded when autophagosomes merge with lysosomes [22]. Using CQ as a lysosomal inhibitor, we showed that LC3B-II levels decreased in KD fibroblasts, demonstrating that autophagic flux was reduced. Finally, we investigated p62/SQSTM1 levels and observed an increased expression during starvation in both control and KD fibroblasts. Among other functions, p62/SQSTM1 is known to be enclosed in autophagosomes and then, to be destined for degradation. Thus, an inverse correlation between p62/SQSTM1 levels and autophagic flux should emerge in this model. However, the employment of p62/SQSTM1 as an indicator of autophagic flux is not universally accepted because of other variables affecting its expression. In particular, it was demonstrated that in starved cells autophagic flux, transcriptional upregulation and availability of lysosomal-derived amino acids concur to define p62/SQSTM1 levels [46]. Moreover, p62/SQSTM1 acts as a scaffold for proteins involved in UPS, signalling, autophagy, and apoptosis [47]. Based on our data, it can be hypothesized that the increase in p62/SQSTM1 transcription could occur in parallel to autophagy in both control and KD fibroblasts during starvation. When CQ was added to block the autophagic flux and autophagosomes degradation, we observed that p62/SQSTM1 accumulation was higher in controls than in KD fibroblasts. Significantly, it was demonstrated that p62/SQSTM1 reduction decreased the recruitment of LC3 to autophagosome during starvation [48]. Summing up, all these data concur to identify impairment in autophagosomes formation and maturation in KD fibroblasts.

To assess the involvement of lysosomes during the final steps of autophagy, we employed the probe Lysotracker red DND-99 and observed the appearance of dilated lysosomes in KD fibroblasts. This finding could be related to lysosomal dysfunction [49]. However, the measurement of lysosomal enzymatic activities, i.e., beta-galactosidase and beta-hexosaminidase, and lysosomal proteins, i.e., LAMP1 and cathepsin D, did not reveal dysfunctions in lysosomal functionality. GALC is a lysosomal enzyme; therefore, its deficiency leads to the impaired metabolism of other lysosomal substrates with increased levels of secondary metabolites [12] in addition to PSY. It is possible to suppose that secondary metabolites could induce lysosomal distress, in particular when autophagy was stimulated. However, we did not observe p62/SQSTM1 or LC3B-II accumulation specifically in KD fibroblasts when CQ was not added to the cells; therefore, we can suppose that the autophagosome degradation step was functioning.

Thus, all data collected proved that autophagic flux was reduced in starved KD fibroblasts and that the steps of autophagosome formation were impaired in KD fibroblasts. We can exclude that these effects could be related to PSY accumulation because we did not observe its presence in KD fibroblasts before and after starvation with CQ addition. However, glycosphingolipid synthesis inhibition through D-PDMP was able to induce LC3B-II increase in KD fibroblasts. We demonstrated that LacCer increase in KD fibroblasts persists also during autophagy induction. This could be particularly relevant because of the association between LacCer increase and AKT/BCL2 upregulation in KD fibroblasts [12]. AKT was demonstrated to be able to inhibit autophagy through different paths including the activation of mTOR and the direct phosphorylation of beclin-1. AKT-phosphorylated beclin-1 strongly interacts with intermediate proteins of the cytoskeleton and shows a reduced capability to trigger autophagy [32]. To demonstrate that AKT upregulation could be responsible for autophagic impairment in KD fibroblasts, we proved that phosphorylation of beclin-1 on Ser295 was enhanced. Furthermore, BCL2 reduces the pro-autophagic action of beclin-1 [50]. In KD fibroblasts, we showed that a higher amount of beclin-1 was associated with BCL2. To confirm these results and the involvement of AKT, the employment of the PI3K inhibitor, LY294002, which in turn reduces also AKT activity and BCL2 expression, was able to reduce Ser295 phosphorylation on beclin-1.

In conclusion, this paper demonstrated that several mechanisms that above all concerning beclin-1 are deregulated in KD fibroblasts. First of all, the induction in beclin-1 increase upon autophagic stimuli is impaired even if basal levels of beclin-1 appear to be the same between control and KD fibroblasts. This evidence is in line with a previous report demonstrating that beclin-1 expression is not altered in several lysosomal storage diseases with normal sphingolipid traffic [51]. Then, inhibitory phosphorylation of beclin-1 and the formation of the complex with BCL2 concur to the impairment of autophagy activation. These findings underline the importance of altered signalling pathways induced by changed levels of secondary substrates in the interpretation of KD pathogenesis. They permit to understand dysfunctions in tissues and cells which are not significantly affected by PSY accumulation or during the early phases of the disease when PSY levels are not so high.

## 4. Materials and Methods

### 4.1. Cell Lines and Treatments

Four different human fibroblast cell lines were used, two from healthy donors (L40 and RB1818) and two from KD patients (VA1679, FO86/78). Cell lines were provided and grown according to Papini et al. [12]. Briefly, L40 fibroblasts were kindly provided by Prof. M. Aureli (University of Milan, Italy), RB1818 and VA1679 fibroblasts were supplied by Dr C. Gellera, Fondazione IRCCS Istituto Neurologico Carlo Besta (Milan, Italy), FFF0861978 (FO86/78) fibroblasts were obtained by the Cell Line and DNA Biobank from patients affected by genetic disease (Gaslini Institute, Genova, Italy), a member of the Telethon Network of Genetic Biobanks [52]. In the text, L40 and RB1818 cells are referred to as Control 1 and Control 2, whereas VA1679 and FO86/78 cells are referred to as Krabbe 1 and Krabbe 2.

To induce autophagy, cell lines were washed once with phosphate-buffered saline (PBS) (8 mM Na_2_HPO_4_, 2 mM KH_2_PO_4_, 137 mM NaCl, 2.7 mM KCl, pH 7.4) and incubated in Earle’s balanced salt solution (EBSS) (5.3 mM KCl, 117.2 mM NaCl, 1.01 mM NaH_2_PO_4_, 1.8 mM CaCl_2_, 0,81 mM MgSO_4_, 26.19 mM NaHCO_3_, 5.56 mM glucose, pH 7.4) for 2 h and 4 h. To study autophagy, cells were treated with 20 μM chloroquine (CQ) (Merck Millipore Darmstadt, Germany) in EBSS for 2 h and 4 h. Non-treated cells (NT) were incubated with the grown medium.

To inhibit glycosphingolipid synthesis fibroblasts were treated with 20 µM d-threo-1-phenyl-2-decanoylamino-3-morpholino-1-propanol (D-PDMP) (Merck KGaA, Darmstadt, Germany) in DMEM plus 10% FBS for 24 h.

### 4.2. Whole-Cell Lysate Preparation

Cells were washed with PBS once and solubilized for 10 min at 4 °C in lysis buffer (25 mM Tris-HCl pH 7.4, 150 mM NaCl, 5 mM EDTA, 1 mM Na_3_VO_4_, 1 mM NaF, 1% *v*/*v* NP40, 10 µg/mL aprotinin, 10 µg/mL leupeptin, 1 µg/mL pepstatin A). After centrifugation at 14,000 g for 10 min at 4 °C, supernatants were collected, and assayed for protein concentration using Coomassie Protein Assay (ThermoFisher Scientific, Waltham, MA, USA). Proteins of interest were analysed by Western blotting.

### 4.3. Western Blotting Analysis

Whole-cell lysates were resolved by SDS-PAGE and electrophoretically transferred to Hybond PVDF membranes (Amersham, GE Healthcare GmbH, Solingen, Germany). PVDF membranes were treated overnight with primary antibodies at 4 °C: anti-SQSTM1/p62 (#5114), anti-Beclin-1 (D40C5), anti-AMPKα (D5A2) (#5831), anti-cathepsin D (E179) (#69854), anti-phospho-Akt (Ser473) (D9E) (#4060), anti-Akt (pan) (C67E7) (#4691) were from Cell Signaling Technology (Danvers, MA, USA); anti-LC3B (L7543), and anti-BCL2 (BCL2-100) (B3170) were from Sigma–Aldrich (Merck KGaA); anti-human LAMP-1 (611042) (clone 25/Lamp-1) was obtained from BD Biosciences (Franklin Lakes, NJ, USA); anti-phospho-Beclin-1 (Ser295) (#PA5-35394) and anti-PBS TFEB (#PA1-9109) were obtained from Invitrogen (ThermoFisher Scientific Waltham, MA, USA). A mouse monoclonal anti-GAPDH antibody (6C5) (MAB-10578) (Immunological Sciences, Roma, Italy) was used as the loading control. After washing, PVDF membranes were incubated with the appropriate secondary antibody conjugate with horseradish peroxidase at room temperature for 1 h. Detection by chemiluminescence and analysis were performed as previously described [12].

### 4.4. Beta-Galactosidase and Beta-Hexosaminidase Enzymatic Activities

26 × 10^4^ cells were plated in 60 mm plates. After 24 h, they were cultivated in EBSS for 4 h and collected by scraping. Cell lysis was performed in PBS plus protease inhibitors by sonication (10 s). Beta-galactosidase activity was assayed by resuspending cell lysates (5 µg) in 50 mM acetate buffer pH 4.5 plus 0.0025% *v*/*v* Triton X-100 (final volume 100 µL) and 500 µM 4-methylumbelliferyl beta-D-galactoside (Sigma-Aldrich), for 10 min at 37 °C. Beta-hexosaminidase activity was assayed resuspending cell lysates (5 µg) in 50 mM citrate buffer pH 4.5 plus 0.0025% *v*/*v* Triton X-100 (final volume 100 µL) and 500 µM 4-methylumbelliferyl-N-acetil-β-D-glucosaminide (Sigma Aldrich), for 10 min at 37 °C. Enzymatic reactions were stopped by adding 25 volumes of 0.2 M glycine pH 10.8. 4-Methylumbelliferone sodium salt (Sigma-Aldrich) was employed for calibration. Enzimatic activity was evaluated by spectrofluorimetric measurement of the 4-Methylumbelliferone released, with excitation at 365 nm and emission at 445 nm, employing NanoDrop ND-3000 (ThermoFisher Scientific). Data were expressed as nmol Methylumbelliferone/mg protein × h.

### 4.5. RNA Extraction and Real-Time PCR

RNA extraction and real-time PCR were performed as previously reported [12]. The sequence of primers was: GAPDH (forward 5′-AGGGCTGCTTTTAACTCTGG-3′; reverse 5′-CATGGGTGGAATCATATTGG-3′); BCL2 (forward 5′-GAGGATTGTGGCCTTCTTTG-3′; reverse 5′-CCCAGCCTCCGTTATCCT-3′).

### 4.6. Fluorescence Microscopy

Autophagosomes formation was monitored by fluorescence microscopy using the fluorescent probe, monodansylcadaverine (MDC) (Santa Cruz Biotechnology, Santa Cruz, CA, USA) (MDC, excitation wavelength 335 nm, emission wavelength 525 nm) and the CYTO-ID^®^ Autophagy detection kit (Enzo Life Sciences, Farmingdale, NY, USA). Lysosomes and acidic vesicles were stained using LysoTracker red DND-99 (Invitrogen, ThermoFisher Scientific Waltham, MA, USA) (excitation wavelength 577 nm, emission wavelength 590 nm). Fibroblasts from healthy donors and KD patients were seeded on glass coverslips in 35 mm plates (4.5 × 10^4^ cells/plate). Cell treatments were performed 48 h after plating. Images were acquired by a fluorescence microscope with a fast high-resolution charge-coupled device camera (Colorview12), using 40× and 100× (oil immersion) objectives (Olympus BX-50, Olympus Europe, Hamburg, Germany). The software for image analysis was analySIS from Soft Imaging System GmbH. The fluorescence intensity associated with single cells present in different fields was evaluated employing the ImageJ software. Data were expressed calculating the mean “corrected (by background subtraction) total cell fluorescence”.

#### 4.6.1. Monodansylcadaverine (MDC) Staining

Fibroblasts were washed in PBS with magnesium and calcium and treated with 20 µM CQ in EBSS for 2 h and 4 h. Non-treated cells were incubated in DMEM + 10% *v*/*v* FBS. At the end of the incubation, cells were stained with 100 µM MDC in PBS with magnesium and calcium + 2% *v*/*v* FBS for 10 min at 37 °C. Then, cells were washed once with PBS with magnesium and calcium + 2% *v*/*v* FBS and coverslips were mounted on glass slides.

#### 4.6.2. CYTO-ID^®^ Autophagy Detection Kit

Cells were washed in PBS with magnesium and calcium and incubated with 20 μM CQ in EBSS for 2 h and 4 h, control cells were incubated with DMEM + 10% *v*/*v* FBS. At the end of the incubation, cells were stained with CYTO-ID^®^ Green detection reagent in PBS with magnesium and calcium + 2% *v*/*v* FBS for 10 min at 37 °C according to the manufacturer’s protocol. Cells were washed once with PBS with magnesium and calcium + 2% *v*/*v* FBS and coverslips were mounted on glass slides.

#### 4.6.3. LysoTracker Red DND-99 Staining

Cells were washed once with PBS and incubated with EBSS for 4h. Control cells were incubated with DMEM +10% *v*/*v* FBS for the same time. At the end of incubation, cells were treated with 50 nM LysoTracker red DND-99 in PBS with magnesium and calcium for 30 min. Then, cells were washed with PBS with magnesium and calcium and coverslips were mounted on glass slides.

### 4.7. Immunoprecipitation

Control and KD fibroblasts were washed once with cold PBS and lysed in lysis buffer (20 mM Tris, pH 7.5, 150 mM NaCl, 1 mM EDTA, 1mM EGTA, 1% *v*/*v* Triton X100, 1 mM NaF, 1 mM Na_3_VO_4_, 10 µg/mL leupeptin, 10 µg/mL aprotinin, 1 µg/mL pepstatin A). Cellular debris was removed by centrifugation at 14,000× *g* for 10 min at 4 °C, and supernatants were collected. An amount of 400 µL of supernatants was mixed with monoclonal antibody anti-BCL2 (Sigma clone Bcl2-100) dilution 1:100, at 4 °C, overnight with gentle agitation. Then, the preformed antibody–antigen complex was mixed with 25 µL of Protein A/G Mix Magnetic Beads (Merck Millipore Darmstadt, Germany) for 30 min at room temperature to capture the immune complex. The beads were washed 3 times with washing buffer (PBS + 0.1% *v*/*v* Tween 20) and the bound proteins were eluted by 0.2 M Glicine-HCl pH 2.5 at room temperature. Immunoprecipitates were neutralized with 1 M Tris-HCl pH 8.5, boiled in sample buffer for native elution and analysed through SDS-PAGE.

### 4.8. KD Fibroblasts Treatment with LY294002

The Calcein AM cell viability assay was performed to set treatment with LY294002 (Cell Signaling Technology, Danvers, MA, USA). Briefly, 48 h after seeding, Krabbe 1 and Krabbe 2 fibroblasts were treated with 20 µM LY294002 for 3 h, 6 h and 24 h in DMEM + 10% *v*/*v* FBS. Control cells were incubated with 0.04% *v*/*v* DMSO. The media were subsequently removed, and adherent cells were washed with PBS and incubated for 30 min at 37 °C in the presence of 2 μM calcein AM (Molecular Probes, ThermoFisher Scientific Waltham, MA, USA) in a medium without FBS. Fluorescence (Ex/Em = 485/530 nm) was measured on a microplate reader (Wallac, VICTOR2, Perkin Elmer Waltham, MA, USA). For Western blotting analyses, VA1679 and FO86/78 fibroblasts were seeded in 60 mm dishes at the density of 1.3 × 10^4^ cells/cm^2^. Treatments were performed 48 h after plating. Cells were treated with 20 µM LY294002 dissolved in DMEM + 10% *v*/*v* FBS for 30 min. Control cells were incubated with 0.02% *v*/*v* DMSO for 30 min. Then, cells were placed in EBSS + 20 μM CQ for 0 h, 3 h, and 6 h. After treatments cells were harvested, whole cell lysates were prepared as described above and analysed by immunoblotting.

### 4.9. Sphingolipid Quantification by LC-MS/MS

Control and KD fibroblasts (100–200 µg proteins) treated for 4 h in EBSS with 20 µM CQ and non-treated were resuspended in PBS (100 µL) and extracted with methanol/chloroform (2:1, *v*/*v*). Sphingolipids were recovered in methanol (100 µL) after being subjected to alkaline methanolysis (75 µL KOH 1 M in methanol), neutralized and evaporated to dryness. The LC-MS/MS consisted of a LC Dionex 3000 UltiMate (ThermoFisher Scientific, Waltham, MA, USA) coupled to a tandem mass spectrometer AB Sciex 3200 QTRAP (AB Sciex, Concord, Canada) equipped with electrospray ionization TurboIonSpray™ source operating in positive mode (ESI+). Chromatographic separation was achieved on a reverse-phase Acquity BEH C8 column 1.7 μm, 2.1 × 100 mm (Waters, MA, USA) equipped with pre-column using as mobile phases (A) water + 0.2% formic acid + 2 mM ammonium formate and (B) methanol + 0.2% formic acid + 1 mM ammonium formate. Five microliters of clear supernatant were directly injected into LC-MS/MS and quantified by multiple reaction monitoring (MRM) [53].

### 4.10. Statistical Analysis

Data were reported as means ± standard deviations (SD) of independent experiments using one-way analysis of variance (ANOVA) followed by Dunnett’s, Tukey’s, or Bonferroni’s multiple comparison tests or using unpaired Student *t*-test to determine significant differences. A *p*-value < 0.05 was considered statistically significant. Data were analysed using the software GraphPad PrismTM 9 (San Diego, CA, USA).

## Data Availability

All data are available from the corresponding author on reasonable request.

## Supplementary Materials

The following supporting information can be downloaded at: https://www.mdpi.com/article/10.3390/ijms24065984/s1, Figure S1: CYTO-ID^®^ Green detection reagent staining of fibroblasts after 2 h in EBSS plus CQ.
