# Supplementary material for: Impaired Autophagy in Krabbe Disease: The Role of BCL2 and Beclin-1 Phosphorylation
Source: Int J Mol Sci. 2023 Mar 22;24(6):5984. doi: 10.3390/ijms24065984 (PMC10051825; doi:10.3390/ijms24065984)

## Slide 1
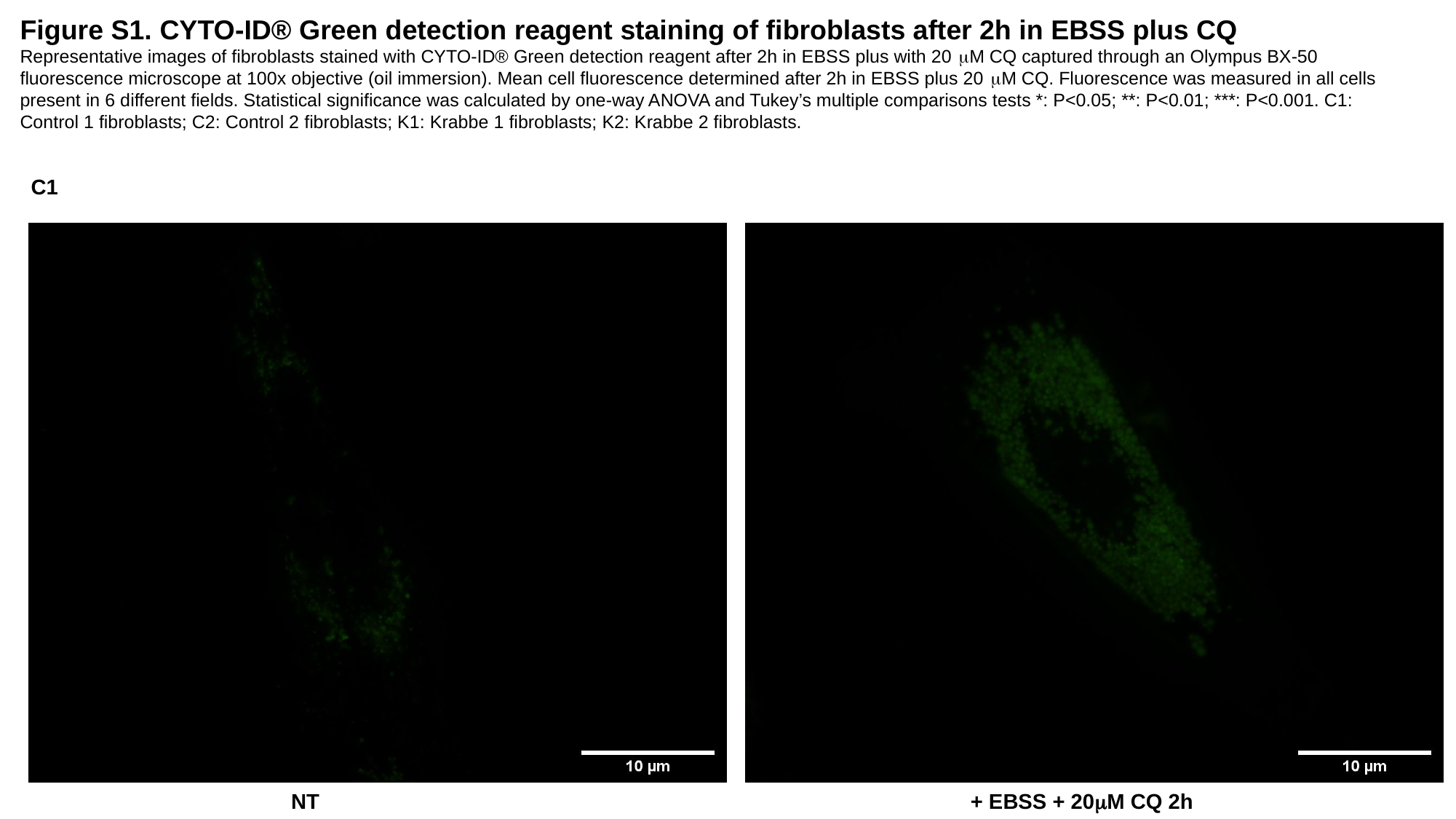

Figure S1. CYTO-ID® Green detection reagent staining of fibroblasts after 2h in EBSS plus CQ
Representative images of fibroblasts stained with CYTO-ID® Green detection reagent after 2h in EBSS plus with 20 μM CQ captured through an Olympus BX-50 fluorescence microscope at 100x objective (oil immersion). Mean cell fluorescence determined after 2h in EBSS plus 20 μM CQ. Fluorescence was measured in all cells present in 6 different fields. Statistical significance was calculated by one-way ANOVA and Tukey’s multiple comparisons tests *: P<0.05; **: P<0.01; ***: P<0.001. C1: Control 1 fibroblasts; C2: Control 2 fibroblasts; K1: Krabbe 1 fibroblasts; K2: Krabbe 2 fibroblasts.
C1
100x
100x
NT
+ EBSS + 20μM CQ 2h

## Slide 2
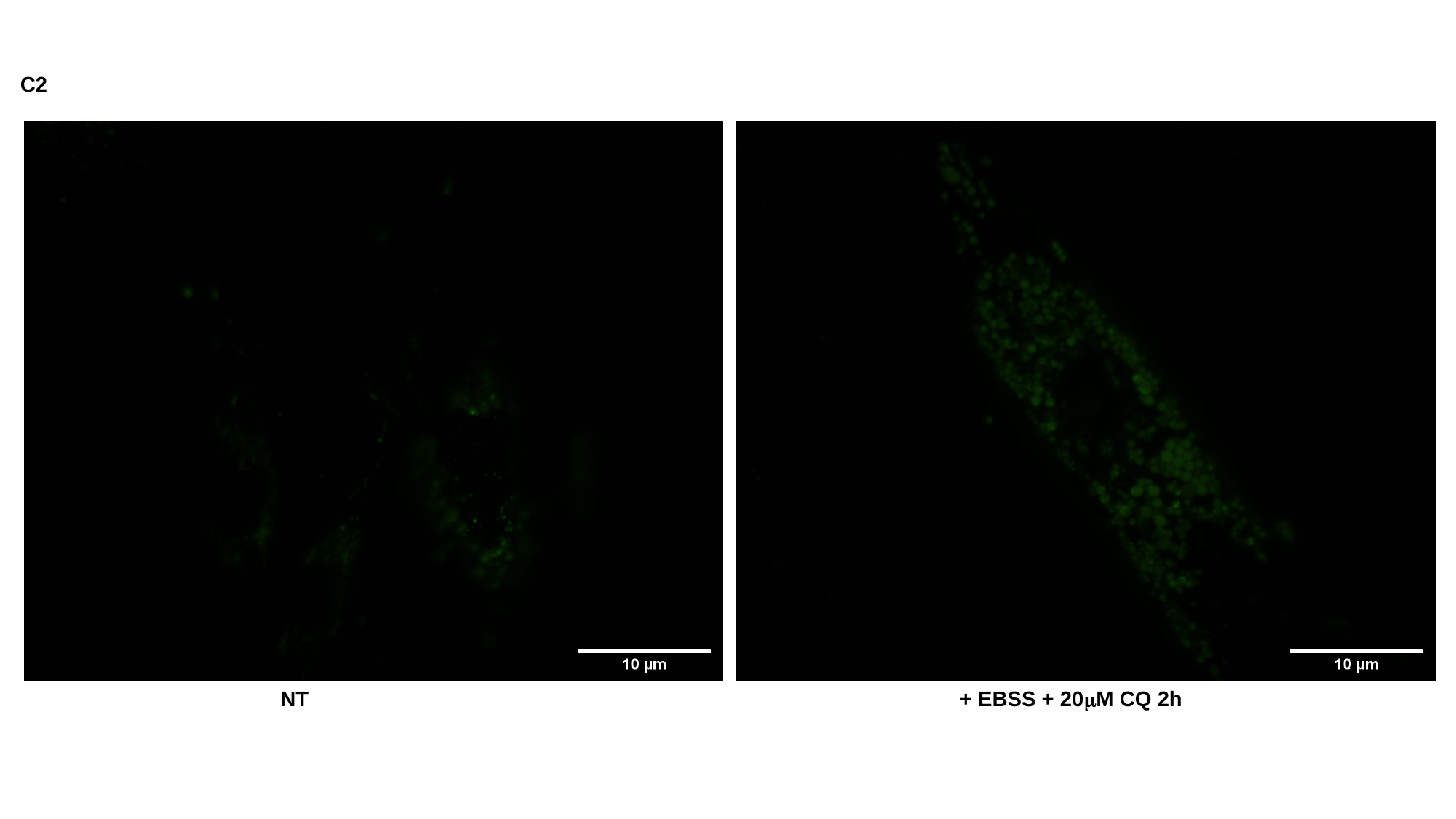

C2
100x
100x
NT
+ EBSS + 20μM CQ 2h

## Slide 3
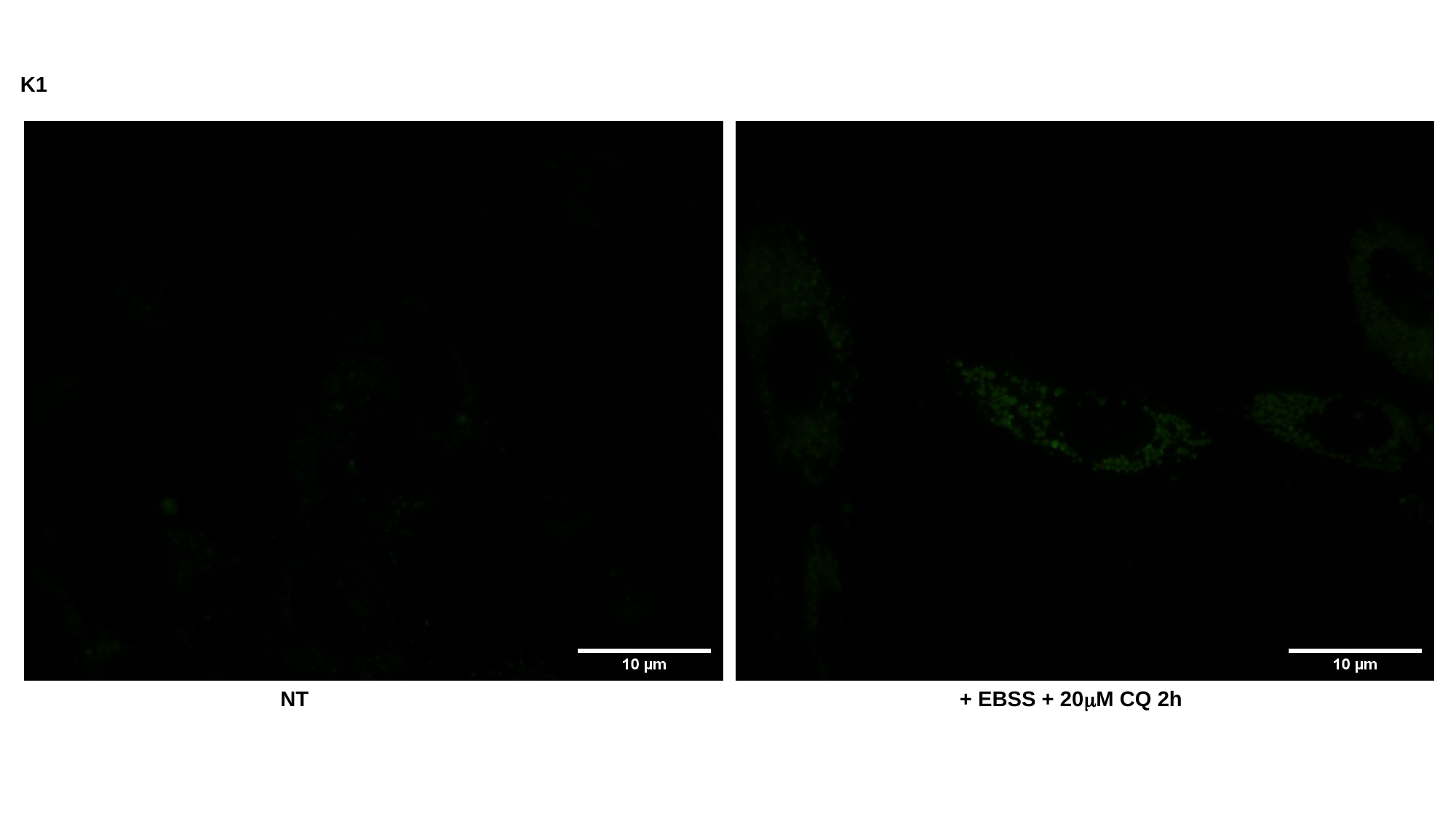

K1
100x
100x
NT
+ EBSS + 20μM CQ 2h

## Slide 4
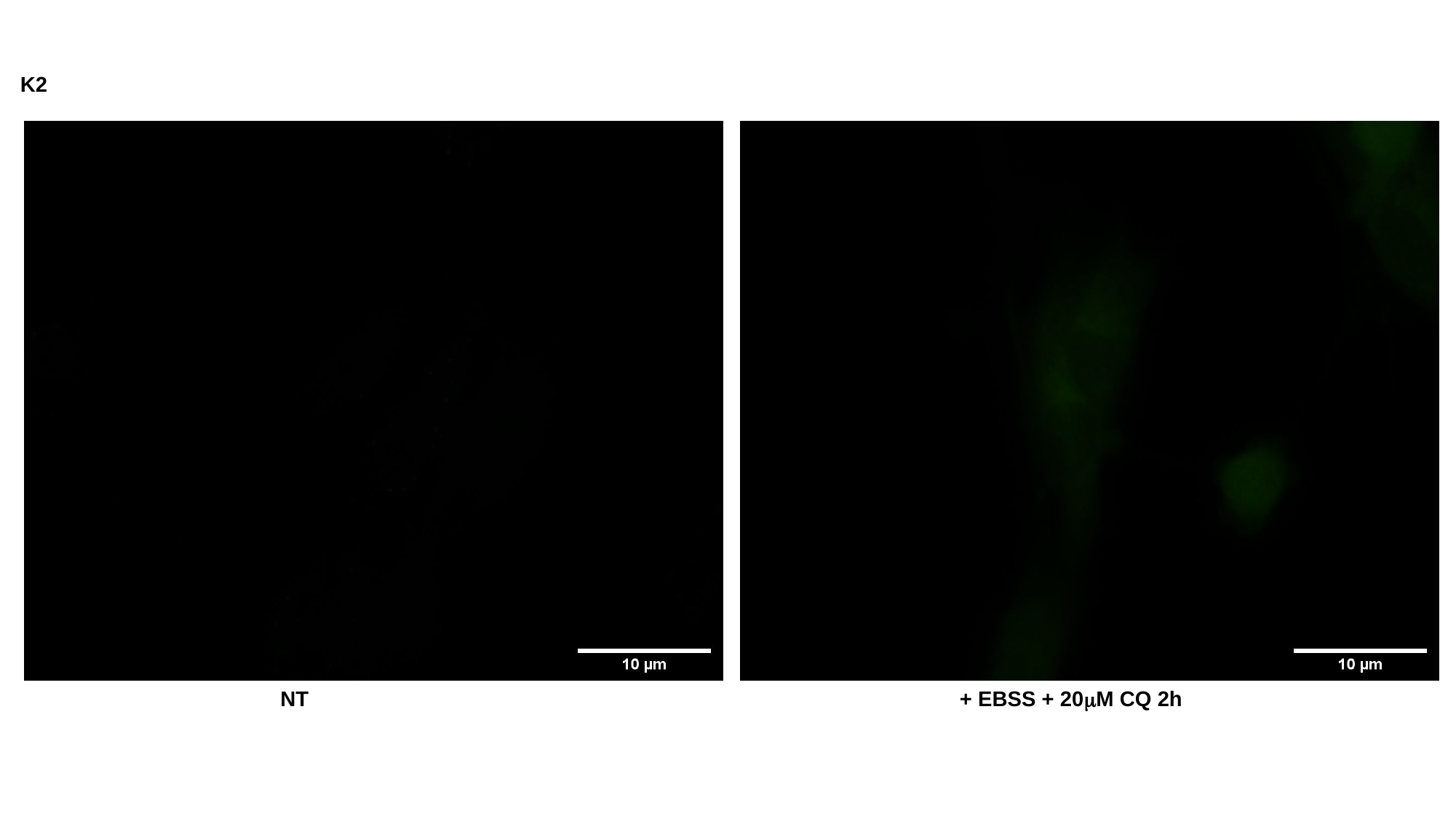

K2
100x
100x
NT
+ EBSS + 20μM CQ 2h

## Slide 5
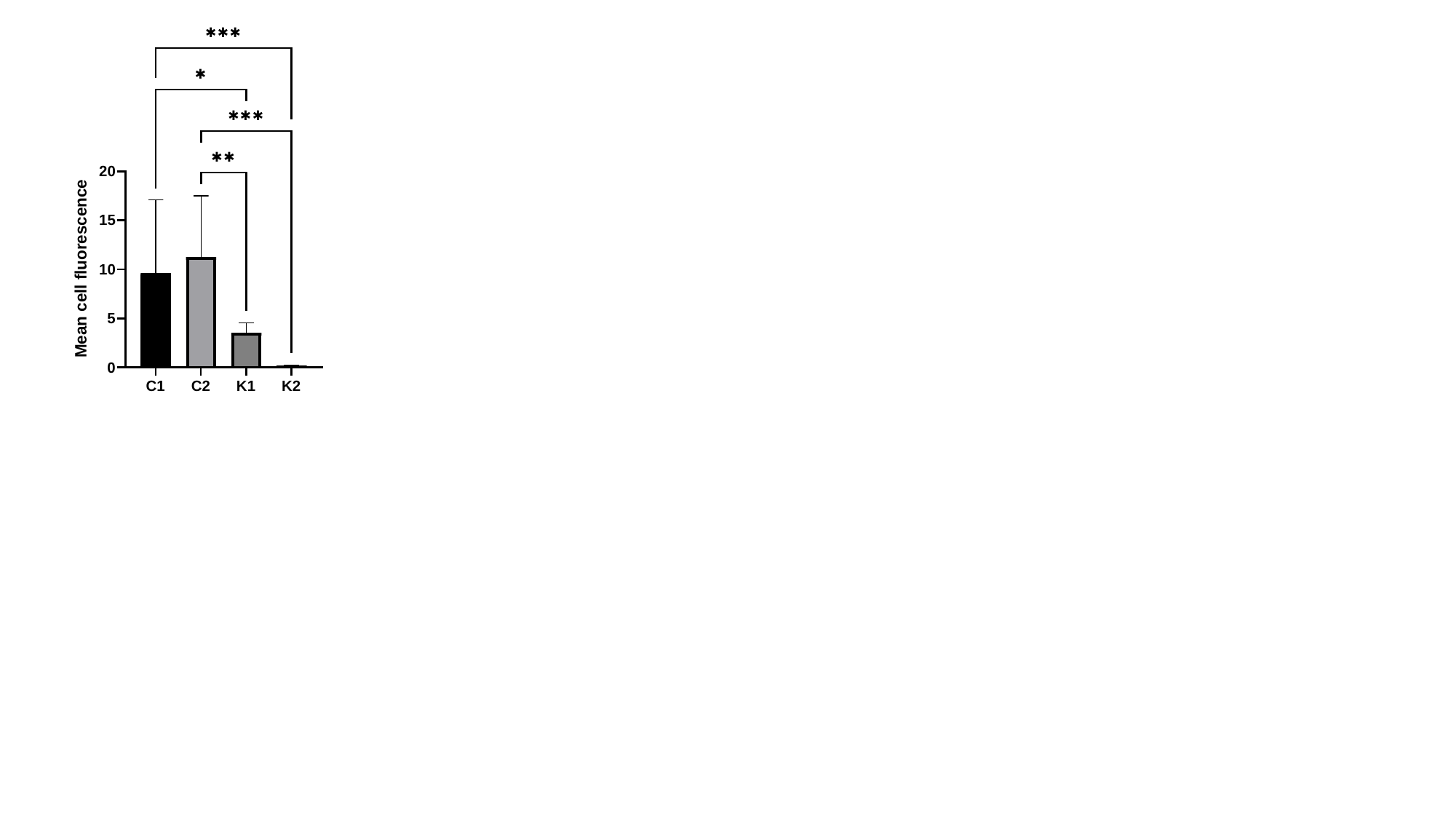

Supplement: Supplementary file 1 [file ijms-24-05984-s001.zip › ijms-2144047-supplementary.pptx]
